# PMCT images of a motorcycle helmet-associated fracture

**DOI:** 10.1007/s12024-017-9911-4

**Published:** 2017-09-05

**Authors:** Christopher Bell, Thomas R. A. Prickett, Guy N. Rutty

**Affiliations:** 1 School of Medicine, University of Liverpool, Liverpool, UK; 2 East Midlands Forensic Pathology Unit, University of Leicester, Robert Kilpatrick Building, Leicester, LE2 7LX UK

## Case 1

**Fig. 1:**
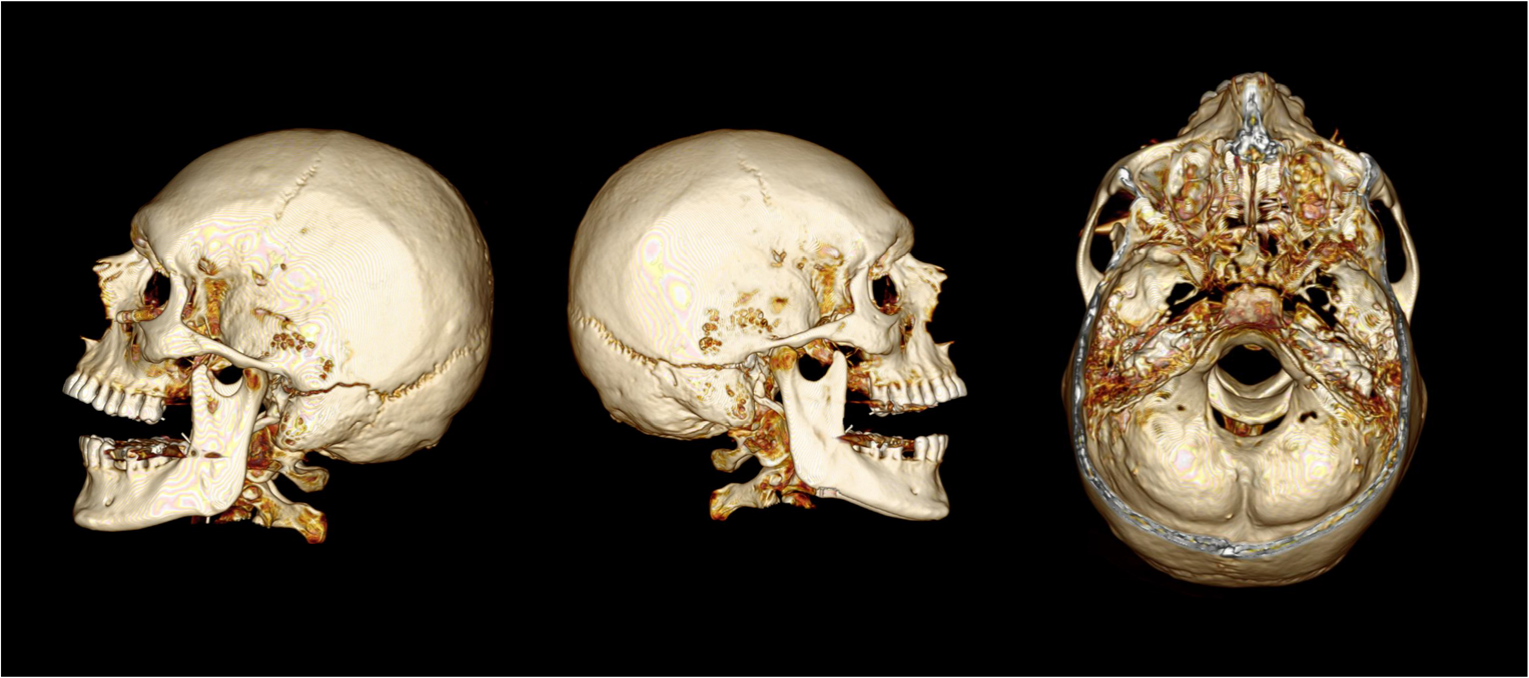
PMCT images of the deceased from case 1, showing views from the left, right, and top (windowed view). The fracture extends through the skull base and continues to the lambdoid sutures bilaterally but does not meet sagittally to completely dissociate the skull portions

A 44 year-old male was involved in a head-on collision with a car which was overtaking another vehicle at the time. After being thrown from the motorcycle the deceased impacted to the car’s nearside, then to the rear offside curtain of an oncoming lorry before coming to rest on the opposite verge of the road. He was declared dead at the scene. The full-face SHOEI brand helmet worn had a witness mark to the front center just above the visor edge. There were multiple areas of bruising and abrasion to the head but no lacerations. Blood was noted to issue from the left ear. Post-mortem computed tomography (PMCT) identified a fracture of the base of the skull centered at the pituitary fossa extending to both middle fossae. It extended to the temporal bone of the vault, and along the lambdoid suture. The fracture did not quite meet at the back of the head, preventing dissociation between two skull portions (Fig. 1).

## Case 2

**Fig. 2:**
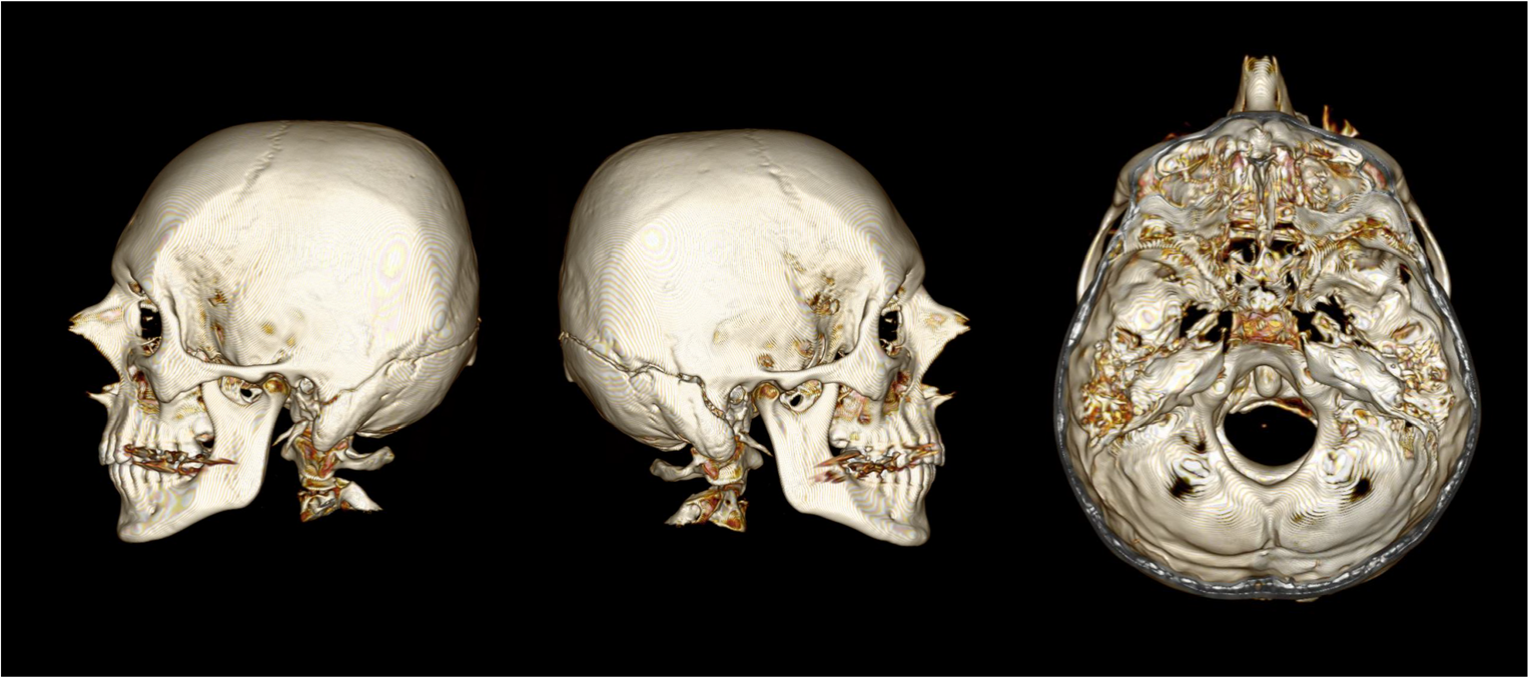
PMCT images of the deceased from case 2, showing views from the left, right, and top (windowed view). In this case the displacement is minimal but there is a clear separation between both portions of the skull

A 40 year-old male was overtaking several vehicles when his motorcycle collided head-on with the front of a car. He was thrown from the motorcycle and landed on the carriageway where he was later pronounced dead. The full-face Arai brand helmet worn had a large vertical crack and scuff marks on the back and damage to the interior padding on the right. The visor was broken with only a small portion remaining attached to the helmet. On external examination there was blood emanating from both ears, bruising to the back of the right ear and a band of red bruising and abrasion to the left chin. PMCT identified a fracture of similar pattern to case 1, but rather than involve the lambdoid suture it extended across the posterior occipital bone to complete a full circumferential fracture and separate the two portions of the skull, with minimal displacement (Fig. 2).

## Case 3

**Fig. 3:**
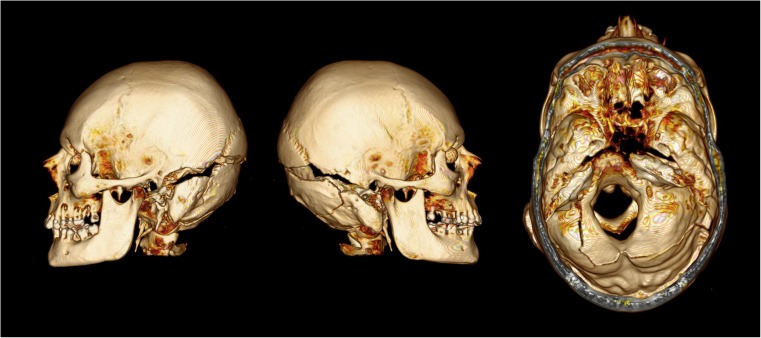
PMCT images of the deceased from case 3, showing views from the left, right, and top (windowed view). There is complete dissociation between the two portions of the skull. An associated fracture extends from the first fracture line around the foramen magnum more akin to a traditional incomplete ring fracture. The deceased had a false right eye

A 51 year-old male was riding his motorcycle at high speed when he collided with the offside passenger door area of a car entering from a side-road. He was thrown in the air somersaulting several times before landing 4–6 m from the car. He was declared dead at the scene. The full-face Shark brand helmet worn had a large vertical crack on the left side extending from the upper edge of the visor upwards. On external examination a closed fracture of the posterior skull was evident as well as bleeding from the right ear and a black eye. PMCT identified a skull fracture of a similar pattern to case 2 with more displacement, and an associated incomplete classic ring fracture of the base of the occiput (Fig. 3).

## Case 4

**Fig. 4:**
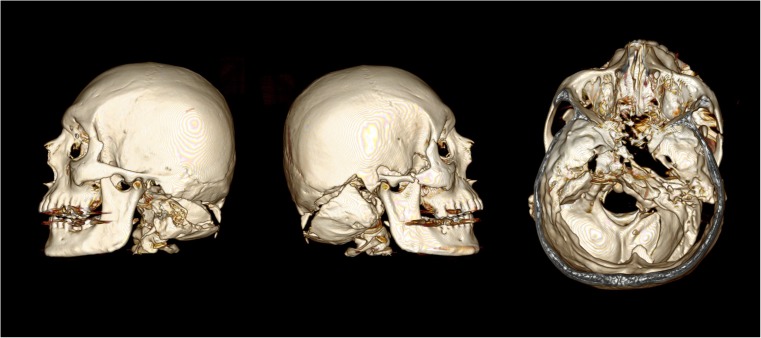
PMCT images of the deceased from case 4, showing views from the left, right, and top (windowed view). There is complete dissociation between the two portions of the skull. In addition portion of the occipital bone below the fracture line is in several pieces

A 37 year-old male was riding his motorcycle which collided with a vehicle travelling in the opposite direction which was performing an overtaking maneuver. He was pronounced dead at the scene. The full-face OGK brand helmet worn had marks around the vent on the face bar of the helmet indicating an impact. The top vent and visor had been broken off. On external examination there was purple bruising and a 12 × 3 cm red abrasion on the underside of the chin, as well as several small areas of bruising and abrasion around the eyes and nose. To the left side of the back of the head was a linear partial thickness laceration measuring 2 cm in length. PMCT identified a fracture of similar pattern to case 2, and below this fracture line the remaining part of the occipital bone had fractured into several pieces (Fig. 4).

## Case 5

**Fig. 5:**
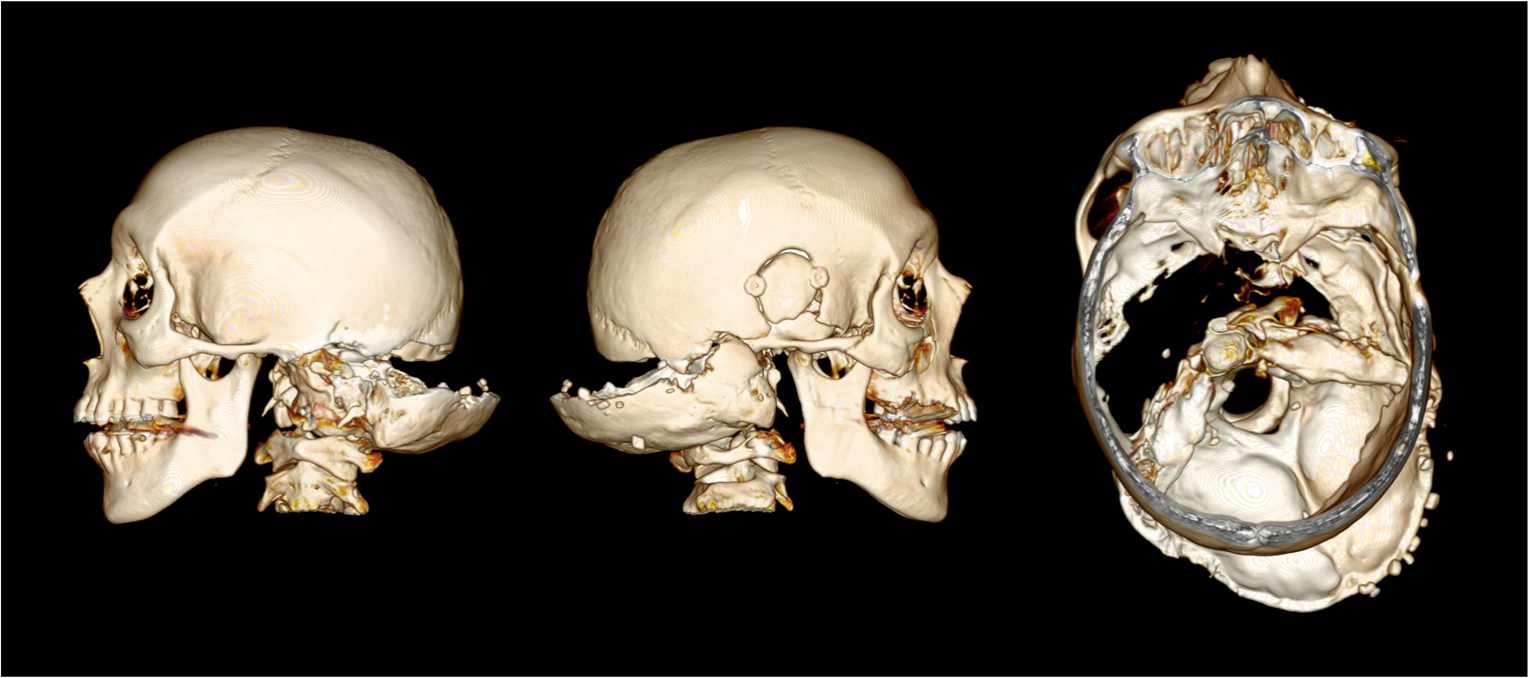
PMCT images of the deceased from case 5, showing views from the left, right, and top (windowed view). There is complete dissociation of the two portions of the skull with significant displacement. Evidence of previous surgery to the right parietal area can be seen

A 42 year-old male was riding his motorcycle when it collided head-on with the passenger door of a vehicle which had skidded onto the opposite side of the road after losing control. It was thought that the motorcycle landed on top of him and the car rolled partially on top of the motorcycle. He was pronounced dead at the scene. The full-face SHOEI brand helmet had a large dented area to the left side of the face bar with a crack in the shell running through it, and the chin strap had marks indicating significant force had been applied through it. On external examination there was a large full thickness laceration extending across the occiput and left face. PMCT identified a fracture of similar pattern to case 2 with massive displacement of the skull parts (Fig. 5).

## Discussion

Post-mortem computed tomography (PMCT) is increasingly being adopted in the investigation of death, usually as an adjunct to autopsy, but increasingly in some countries to replace the internal examination [1]. It has been reported to have an important role in the investigation of trauma due to its ability to investigate skeletal injury [2]. In this context it has been shown to be useful in motorcycle fatalities [3].

Motorcyclists remain some of the most at-risk road users worldwide. In the United Kingdom motorcyclists are 68 times more likely to suffer a fatality on the road per passenger mile than car occupants [4]. Although helmet use has been shown to be effective in reducing injuries to the head, head injury is still the leading cause of death among both helmeted and non-helmeted riders [5, 6]. As these are closed injuries the extent of the head injury may not be appreciated by those rendering aid at the roadside. Despite the clear benefit of wearing a helmet, there appear to be predictable patterns of skull fracture associated with certain types of helmet impacts. Cooter et al. reported a fracture of the middle cranial fossa traversing the clivus believing that an impact to the face-bar transmitted a force to the skull base via the mandibular condyles, a mechanism also considered by Harms [7, 8]. Heavier helmets are more likely to result in a partial or complete ring fracture of the base of the skull when subjected to axial loading [9].

The five cases presented here demonstrate the same fracture pattern: a circumferential fracture comprising of a linear fracture of the middle cranial fossa extending through the pituitary fossa, continuing to the temporal and occipital bones of the skull vault. They are presented in order of increasing severity. Case 1 was less severe without dissociation of the skull. In cases 2–5 the fracture was sufficiently severe to completely dissociate the skull into two parts. Case 3 had an additional associated defect more akin to a classic incomplete ring fracture of the skull base. Cases 2, 4 and 5 show the fracture-type largely alone. Of note all of the deceased had witness marks on their helmet indicating an impact to the face, except for the individual in case 2, who clearly had an impact to the chin from the injuries noted on examination. All died at the scene due to their head injuries, although several had other injuries to the chest or abdomen which contributed equally to death.

A ring fracture is classically caused by an impact either to the top of the head or a fall from a height landing feet first, and involves the petrous temporal bones bilaterally, the clivus and the posterior part of the foramen magnum [10, 11]. However, the pattern described here also bears resemblance to a hinge (or ‘motorcyclist’s’) fracture in which a transverse crack extends the width of middle cranial fossa through the pituitary fossa [10]. Krantz briefly described a fracture which may have similar features to our series, which resulted from ‘a force which lifts the upper part of the head from the base of the skull’ [12]. The majority of these cases suffered an impact to the face. The lack of detail in these studies’ descriptions make it difficult however to confirm the similarity in the fracture pattern. Moskała et al. included an image of a ‘circular’ fracture in their study of PMCT in motorcyclists which shares the features in our cases; it is not further described, except to note that these types of fractures can be difficult to see on traditional autopsy [3]. A review of basal skull fractures in relation to helmet-use also described several fractures with features similar to our cases [13]. West et al. also demonstrated fractures extending from the skull base to the occipitomastoid suture (as in case 1), but these were not bilateral and did not involve full circumferential fracture of the occipital bone seen in cases 2–5 [14]. The fracture pattern presented here has features of both a hinge and a ring fracture. Cases 2, 4 and 5 had clear evidence of an impact to the chin area, which could cause the fracture via force through the mandible, as described in the literature [7, 8]. However although this may be the most frequent cause it is thought that skull base and vault fractures can result from impacts anywhere on the head [13, 15].

We suggest that the fractures presented in the five cases here are distinctly associated with motorcycle incidents. Similar fractures may have been described briefly within larger studies, and they may be familiar to experienced autopsists, however to our knowledge, this is the first time the fracture has been described in detail and as a series illustrated using PMCT.
